# Humanitarian Love and Ethical Dilemmas in Mental Health Care: A Literature Review

**DOI:** 10.1192/j.eurpsy.2025.1563

**Published:** 2025-08-26

**Authors:** A. L. Batiridou, E. Dragioti, S. Mantzoukas, M. Gouva

**Affiliations:** 1 Research Laboratory of Psychology of Patients, Families and Health Professionals, School of Health Sciences, Department of Nursing; 2 Research Laboratory of Integrated Health, Care, and Well-being, School of Health Sciences, Department of Nursing, University of Ioannina, Ioannina, Greece

## Abstract

**Introduction:**

Humanitarian love, a concept grounded in compassion, emotional support, and empathy, plays a pivotal role in mental health care, where patients often confront profound emotional and psychological challenges. This compassionate approach strengthens patient-centered care by fostering deeper therapeutic relationships between mental health professionals and patients. However, balancing this emotional engagement with professional responsibilities can give rise to complex ethical dilemmas.

**Objectives:**

This literature review aims to: a) examine how humanitarian love influences mental health practice, and b) explore the ethical tensions between respecting patient autonomy and ensuring beneficence.

**Methods:**

A literature review of scientific articles was conducted using databases such as PubMed, Scopus, and Cochrane, with a focus on recent research exploring the role of humanitarian love in mental health care.

**Results:**

The findings suggest that humanitarian love enhances the quality of mental health care by promoting empathy, trust, and greater patient engagement. However, significant challenges arise when mental health professionals must navigate the balance between compassion and their ethical and legal obligations. The assessment of a patient’s decision-making capacity is inherently subjective, which can potentially lead to bias or burnout among mental health professionals. Furthermore, cultural factors often add complexity to mental health care, particularly in global or cross-cultural contexts. In such situations, well-intentioned humanitarian interventions may inadvertently conflict with local traditions and human values.

**Conclusions:**

While humanitarian love is essential in mental health care, it frequently comes into conflict with the ethical principles of autonomy and beneficence. The subjective nature of assessing a patient’s decision-making capacity, combined with cultural influences, requires mental health professionals to adopt a sensitive and nuanced approach to ensure balanced and high-quality healthcare services.

**Disclosure of Interest:**

None Declared

